# Expression of 10 circulating cytokines/chemokines in HBV-related liver disease

**DOI:** 10.1186/s13027-024-00580-9

**Published:** 2024-05-01

**Authors:** Yanfang Jia, Xiaolei Jiao, Wenxia Shi, Ying Luo, Huiling Xiang, Jing Liang, Yingtang Gao

**Affiliations:** 1 The Third Central Clinical College of Tianjin Medical University, Tianjin, 300170 China; 2 Tianjin Key Laboratory of Extracorporeal Life Support for Critical Diseases, Tianjin Institute of Hepatobiliary Disease, Nankai University Affiliated Third Center Hospital, Jintang Road 83#, Hedong District, Tianjin, 300170 China; 3 Department of Gastroenterology and Hepatology, Nankai University Affiliated Third Center Hospital, Tianjin, 300170 China

**Keywords:** Liver disease, Hepatitis B virus, Chronic hepatitis B, Circulating cytokines, Chemokines, Multiplex assays

## Abstract

**Background:**

Cytokines/chemokines play essential roles in the occurrence and progression of hepatitis B virus (HBV) infection. This study aimed to observe the expression patterns of 10 related cytokines/chemokines in the serum of healthy individuals, self-limited patients and HBV-infected patients at different stages of disease (chronic hepatitis B (CHB), liver cirrhosis (LC), hepatocellular dysplastic nodules (DNs) and hepatocellular carcinoma (HCC)) and to analyze the relationships of these cytokines/chemokines with disease progression.

**Methods:**

The levels of six cytokines (FGF-2, IFN-α2, IL-4, IL-6, IL-10 and VEGF-A) and four chemokines (GRO-α, IL-8, IP-10 and MCP-1) were quantified using Luminex multiplex technology.

**Results:**

There were no significant differences in the expression of the 10 cytokines/chemokines between healthy individuals and self-limited patients. The levels of IL-4, IL-6, and IL-8 increased significantly in the CHB and LC groups. IL-10 was highly expressed in the HCC group. The level of IP-10 was significantly greater in all liver disease groups (CHB, LC, DN and HCC) than in the HI and SL-HBV groups, while the level of GRO was significantly lower in all liver disease groups than in the HI and SL-HBV groups. The levels of the 10 cytokines/chemokines were not significantly different between the preoperative group and the two-day postoperative group. Significant increases in the levels of IL-4, VEGF-A and IL-8 and significant decreases in those of IL-10 and GRO-α were observed 3 months after surgery. Correlation analysis revealed that most of the cytokines/chemokines with significant correlation differences were positively correlated before and after HCC surgery.

**Conclusion:**

Our results highlight the fluctuating status of specific cytokines in HBV infection-related disease progression. It is speculated that these cytokines may be used as serum markers to monitor dynamic changes during the progression of HBV-related liver disease and to predict patient prognosis.

## Introduction

Hepatitis B virus (HBV) infection affects approximately 250 million people worldwide, and there are 500,000 to 700,000 deaths each year due to complications caused by HBV infection [1]. Chronic HBV infection leads to activation of the compensatory mechanism for liver cell death by triggering a sustained inflammatory response, which gradually worsens to cirrhosis and eventually to hepatocellular carcinoma (HCC) [2, 3]. Accumulated evidence suggests that the development of HCC is closely related to the tumor microenvironment (TME), and cytokines/chemokines produced by immune cells and cancer cells are critical regulators of the TME [4, 5]. Inflammatory cells infiltrate the liver and participate in tumor development and progression by stimulating an immune response. Notably, several cytokines/chemokines play essential roles in the pathogenesis, progression, invasion, and metastasis of HCC that progresses from chronic hepatitis B (CHB) [5–7].

Unfortunately, HCC is often diagnosed in the middle or late stage, and the recurrence rate is as high as 70% [8]. Therefore, serological detection of HBV-related liver disease is critical for monitoring progression and determining prognosis. Previous studies have investigated the role of serum cytokine detection in HBV-associated liver disease [9, 10]. Most related studies have focused on the effect of only a single cytokine, but the regulatory network of cytokines is highly complex [2, 11]. This complexity suggests that only simultaneous and consistent measurements of serum cytokine concentrations and a comprehensive understanding of how cytokines are related to each other will help define their roles in tumor development [2, 12, 13]. Cytokines are also structurally pleiotropic, making specific targeting of critical cytokines extremely difficult [11]. Ideal tumor markers should have strong tumor sensitivity and specificity. In recent years, to improve the percentage of positive cells, serological detection of tumor markers has developed in the direction of combined screening for multiple markers. The purpose of this study was to determine the expression patterns of 10 cytokines or chemokines in different stages of HBV-related liver disease and explore candidate biomarkers for determining the prognosis of HCC patients.

## Materials and methods

### Patient selection

This was a retrospective case‒control study. According to the diagnostic criteria of the Guidelines for the Prevention and Treatment of Chronic Hepatitis B (version 2015) and the Specifications for Diagnosis and Treatment of Primary Liver Cancer (version 2017), 106 individuals from the biological sample bank of Tianjin Third Central Hospital were included. This study included 20 healthy individuals (HI group), 11 self-limited hepatitis B virus (SL-HBV) infection patients (SL-HBV group), 20 CHB patients (CHB group), 18 hepatitis B virus liver cirrhosis (LC) patients (LC group), 18 hepatocellular dysplastic nodule (DN) patients (DN group; pathological diagnosis based on biopsy, including eight patients with low-grade dysplastic nodules and ten with high-grade dysplastic nodules) and 19 HCC patients undergoing radical resection (HCC group). One blood sample was collected from each patient in the HCC group before surgery, one blood sample was collected at two days post surgery, and one blood sample was collected at three months post surgery. All HCC patients were given cephalosporins (first or second generation) within 2 days after surgery, and there was no recurrence after a 3-month follow-up. The subjects in each group were of Han nationality in the Tianjin area. The routine blood and biochemical indices of the healthy individuals were normal, and the HBV immunological marker results were negative. The SL-HBV-infected patients had evidence of HBV infection (positive for at least two of the anti-HBs, anti-HBe, and anti-HBc antibodies; negative for HBsAg, HBeAg, and HBV-DNA); had a normal liver function indicator; had no history of liver disease or a clear history of acute hepatitis B; and had recovered without any treatment. The exclusion criteria included coinfection with other viruses, such as hepatitis A virus, hepatitis C virus, or hepatitis D virus, and other liver diseases, including alcoholic liver disease, metabolic liver disease, fatty liver disease, and liver fibrosis. All blood samples in this study were collected and processed with patient consent, and the study was approved by the Ethics Committee of Tianjin Third Central Hospital (IRB2019-040-01).

### Biochemical parameters and virological detection

Parameters of liver function, including ALT, AST, AFP, AFU, TBIL, ALB, GLO, and the white-to-sphere ratio (A/G), were measured by a Roche automatic biochemical analyzer (Roche Cobas c 701; Roche, Switzerland). The serum HBV-DNA load was quantitated using an automatic nucleic acid purification and fluorescence PCR analysis system (Anadas9850, Amplly, China). The levels of HBsAg, anti-HBs, HBeAg, and anti-HBe were tested using an automatic microparticle chemiluminescence immune analyzer (ARCHITECT i4000SR, Abbott, USA). HBsAg negativity was defined as < 50 IU/ml.

### Cytokine measurements

Fasting peripheral venous blood (3 ml) was collected from the subjects, and the serum was isolated and stored at -80 °C for cytokine detection. EMD Millipore’s MILLIPLEX® MAP Human Cytokine/Chemokine Magnetic Bead Panel (Cat. # HCYTOMAG-60 K; EMD Millipore, Billerica, MA, USA) was used for the simultaneous quantification of six human cytokines (fibroblast growth factor 2 (FGF-2), interferon-α2 (IFN-α2), interleukin-4 (IL-4), interleukin-6 (IL-6), interleukin-10 (IL-10) and vascular endothelial growth factor A (VEGF-A)) and four chemokines (growth-related oncogene-alpha (GRO-α/CXCL1), interleukin-8 (IL-8/CXCL8), interferon-γ-inducible protein 10 (IP-10/CXCL10) and monocyte chemotactic protein 1 (MCP-1/CCL2)). The experimental procedure was carried out according to the manufacturer’s protocol. Briefly, the main steps included preparing serum samples and immunoassay reagents, performing the immunoassay procedure, washing the plate, setting up equipment settings and on-machine storage, and analyzing average fluorescence intensity data. Each experiment was performed in duplicate using the same procedure. The analysis step was performed using a Luminex 200 System with xPONENT® software (Luminex, Austin, TX, USA).

### Statistical analysis

Descriptive statistics were calculated to characterize the study population. Serum HBV-DNA levels and HBsAg concentrations were logarithmically transformed for analysis. Categorical variables are expressed as the absolute and relative frequencies, and comparisons between groups were performed using the chi-square test or Fisher’s exact test. Continuous variables with a normal distribution are expressed as the mean ± standard deviation (SD). One-way analysis of variance was used to compare the means of multiple samples. Continuous variables with a nonnormal distribution are expressed as medians (Q1, Q3), and the Mann‒Whitney U test and Kruskal‒Wallis test were used to compare two groups or multiple groups. A two-tailed *p* value less than 0.05 was considered to indicate statistical significance. A Spearman correlation matrix was used to analyze the correlations of the 10 cytokines/chemokines. Correlations with a *p* value < 0.05 were included in the network visualization. The statistical analyses were performed using SPSS version 26.0 (SPSS, Inc., Chicago, IL, USA) and GraphPad Prism 8.0 (GraphPad Software, La Jolla, CA, USA).

## Results

### Patient demographics and clinical characteristics

In this study, 106 subjects (66 males and 40 females), including 20 individuals in the HI group, 11 in the SL-HBV group, 20 in the CHB group, 18 in the LC group, 18 in the DN group, and 19 in the HCC group, were included; their mean age was 52.32 ± 12.82 years. There were significant differences in the levels of ALT, AST, AFP, TBIL, ALB, and GLO and in the A/G among the HI, SL-HBV, CHB, LC, DN, and HCC groups. There were significant differences in the HBV-DNA load, HBsAg level, HBeAg content, and AFU level among the CHB, LC, DN, and HCC groups. GP73 levels and Child‒Pugh stage were significantly different among the LC, DN, and HCC groups. The demographic and clinical characteristics of all patients are summarized in Table 1.

**Table 1:** Demographics and clinical characteristics of all subjects

Characteristics	HI group (*n* = 20)	SL-HBV group (*n* = 11)	CHB group (*n* = 20)	LC group (*n* = 18)	DN group (*n* = 18)	HCC group (*n* = 19)	Statistical values	*P*
Male/female, n	10/10	8/3	10/10	8/10	15/3	15/4	11.157^+^	0.048
Age (years), mean ± SD	46.75 ± 11.47	51.91 ± 10.80	44.95 ± 16.73	56.94 ± 9.95	59.06 ± 10.67	54.42 ± 9.34	4.364^†^	0.001
ALT (U/L), median (Q1, Q3)	21.50 (12.50,27.50)	21.00 (14.00,37.00)	123.00 (20.75,935.25)	24.00 (20.50,49.25)	49.00 (26.50,70.75)	37.00 (24.00,55.00)	24.418^*^	<0.001
AST (U/L), median (Q1, Q3)	21.00 (18.00,26.00)	26.00 (18.00,32.00)	90.50 (23.75,370.20)	23.50 (13.50,63.75)	41.50 (23.50,79.50)	40.00 (27.00,59.00)	25.059^*^	<0.001
AFP (ng/ml), median (Q1, Q3)	2.22 (1.41,3.28)	2.19 (1.48,2.88)	17.00 (9.67,71.74)	9.69 (4.44,37.34)	20.69 (5.68,59.09)	167.72 (10.32,416.55)	60.967^*^	<0.001
TBIL (µmol/L), median (Q1, Q3)	13.15 (11.23,16.90)	20.10 (16.20,23.40)	43.65 (9.70,150.88)	22.20 (17.08,41.95)	15.75 (14.05,19.08)	14.30 (11.20,20.60)	17.142^*^	0.004
ALB (g/L), median (Q1, Q3)	47.25 (45.48,48.80)	43.90 (36.00,50.50)	47.75 (42.10,49.35)	30.40 (28.38,35.50)	34.65 (30.80,39.75)	41.30 (37.60,45.50)	55.690^*^	<0.001
GLO (g/L), median (Q1, Q3)	27.90 (24.78,30.15)	25.20 (23.00,32.20)	29.40 (27.23,34.60)	33.65 (27.75,39.03)	29.55 (27.63,31.13)	30.10 (26.20,32.10)	12.007^*^	0.035
A/G, mean ± SD	1.74 ± 0.26	1.64 ± 0.36	1.54 ± 0.36	0.97 ± 0.25	1.22 ± 0.22	1.38 ± 0.30	16.628^†^	<0.001
HBV-DNA (log10 IU/ml), median (Q1, Q3)	Neg.	Neg.	4.75 (3.31,7.34)	5.38 (3.40,6.32)	1.85 (1.68,1.93)	4.34 (2.78,5.30)	41.103^*^	<0.001^‡^
HBsAg (log10 IU/ml), median (Q1, Q3)	Neg.	Neg.	3.95 (3.42,4.21)	2.46 (2.34,2.74)	2.34 (1.63,3.02)	2.39 (2.05,2.42)	32.487^*^	<0.001^‡^
HBeAg (PEIU/ml), median (Q1, Q3)	Neg.	Neg.	1019.83 (782.12,1551.09)	687.77 (47.89,1187.27)	410.69 (7.90,1218.45)	190.43 (50.20,380.46)	24.465^*^	<0.001^‡^
ALP (U/L)	NA	NA	NA	83.60 (75.50-94.25)	97.75 (69.75-112.75)	88.00 (83.00-124.00)	4.244^*^	0.120
AFU (23U/U), median (Q1, Q3)	NA	NA	42.50 (36.00,54.75)	27.50 (19.50,36.75)	31.50 (25.75,39.25)	37.00 (26.00,46.0)	12.145^*^	0.007^‡^
GP73 (ng/mL), mean ± SD	NA	NA	NA	156.27 ± 52.81	114.05 ± 70.41	95.74 ± 67.24	4.316^†^	0.018^※^
Child‒Pugh stage, (A/B/C)	NA	NA	NA	4/11/3	12/5/1	17/2/0	17.826^*^	<0.001^※^

ALP: alkaline phosphatase; ALT: alanine aminotransferase; AST: aspartate aminotransferase; AFP: alpha-fetoprotein; AFU: alpha-L-fucosidase; TBIL: total bilirubin; ALB: albumin; GLO: globulin; A/G: albumin-globulin ratio; GP73: Golgi glycoprotein 73; HBV-DNA: hepatitis B virus DNA; HBsAg: hepatitis B surface antigen; HBeAg: hepatitis B envelope antigen; Child‒Pugh stage: grading standards for assessing the severity of liver dysfunction; NA: not applicable; Neg. : negative; SD: standard deviation^+^Chi-square test; ^†^One-way analysis of variance; ^*^Kruskal‒Wallis test. ^‡^Comparison among the CHB, LC, DN and HCC groups. ^※^ Comparison among the LC, DN and HCC groups.

### Reliability and accuracy analysis of test data

Due to the low content of some samples, we used twice-repeated fluorescence intensity (MFI) data to calculate and analyze cytokine concentrations via 5- and 3-parameter logistic methods. For samples with a lower content, the five-parameter analysis gave a result “less than the lower limit”, while the three-parameter analysis gave specific values. Statistical analysis revealed that the two algorithms were the same for the four chemokines with high expression levels (GRO-α, IL-8, IP-10, and MCP-1). Although there were many samples with IL-6 and IL-10 concentrations at the lower detection limits (1.69 and 2.45, respectively), the lower detection limits of the two experiments were the same, so the results were not significantly different. However, there were many samples with FGF-2, IFN-α2, IL-4, and VEGF-A concentrations at the lower detection limits, and the lower detection limits were significantly different, resulting in significant differences in the data from the two experiments. After combining the two experimental datasets, three- and five-parameter analyses were performed. The results for six cytokines/chemokines (IL-6, IL-10, GRO-α, IL-8, IP-10, and MCP-1) were consistent and reliable, while those for four cytokines (FGF-2, IFN-α2, IL-4, and VEGF-A) were different; moreover, the results of the three-parameter analysis were relatively more reliable.

### Expression levels of the 10 cytokines/chemokines in the HI group and HBV infection groups

**Fig. 1:**
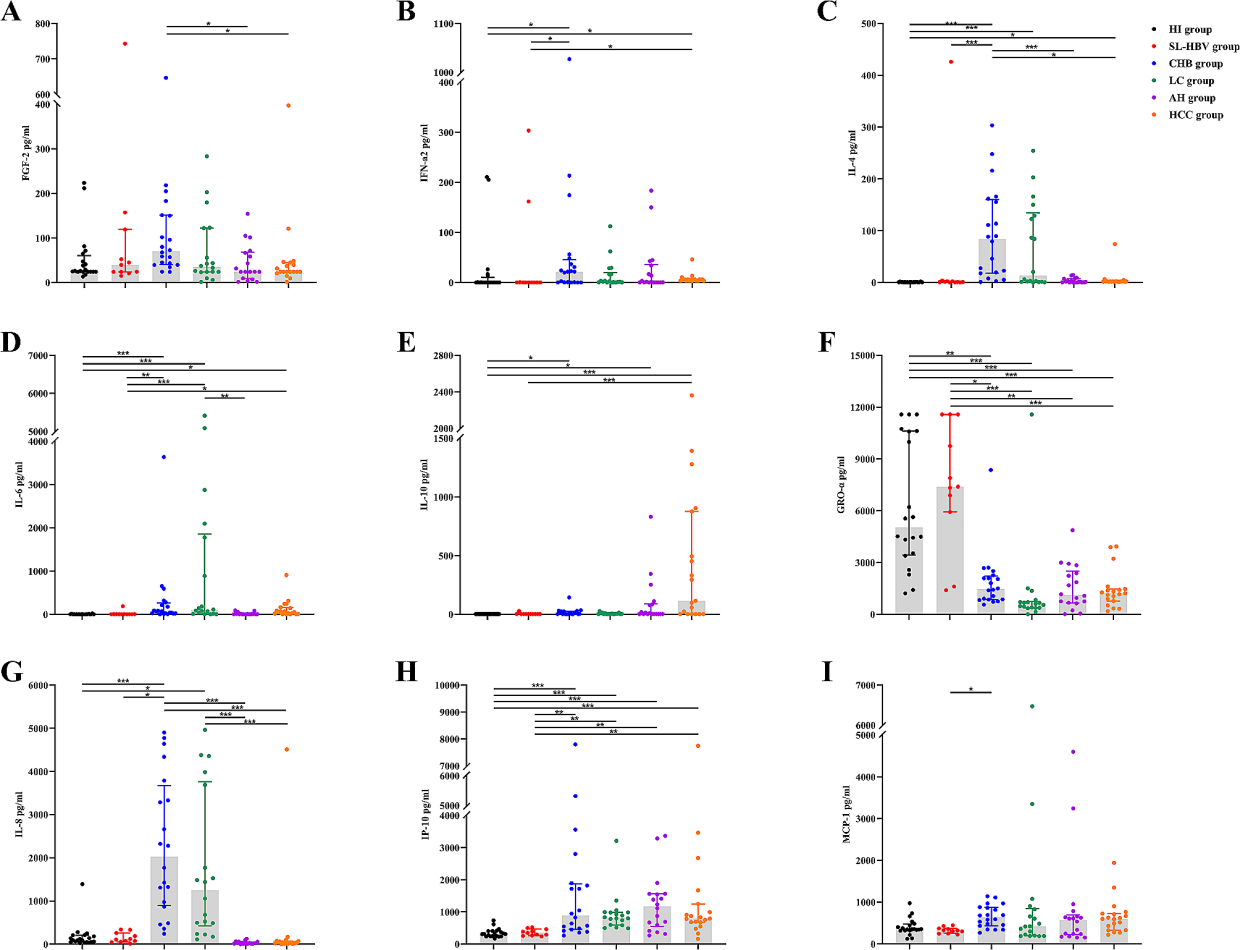
Expression of nine cytokines/chemokines in the HI, SL-HBV, CHB, LC, DN and HCC groups. The nine cytokines/chemokines included FGF-2, IFN-α2, IL-4, IL-6, IL-10, GRO-α, IL-8, IP-10, and MCP-1. (* *P* < 0.05; ** *P* < 0.01; *** *P* < 0.001)

As shown in Table 2; Fig. 1, the serum expression levels of the 10 cytokines/chemokines were not significantly different between the HI group and the SL-HBV group. Except VEGF-A, the other 9 cytokines were significantly different (*P* < 0.05) among the four liver disease groups and between the liver disease groups and the HI/SL-HBV group. FGF-2 expression in the CHB group was significantly greater than that in the DN and HCC groups (*P* = 0.023; *P* = 0.013) (Fig. 1A). The expression of IFN-α2 in the CHB and HCC groups was significantly greater than that in the HI (*P* = 0.030; *P* = 0.050) and SL-HBV groups (*P* = 0.029; *P* = 0.045) (Fig. 1B). The expression of IL-4 in the CHB group was the highest, and there were significant differences between the CHB group and the HI, SL-HBV, DN and HCC groups (*P* = 0.000; *P* = 0.001; *P* = 0.000; *P* = 0.022) (Fig. 1C). In addition, the expression of IL-4 in the LC and HCC groups was greater than that in the HI group (*P* = 0.000; *P* = 0.040) (Fig. 1C). The expression of IL-6 in the LC group was the highest, followed by that in the CHB and HCC groups, which significantly differed from that in the HI (*P* = 0.000; *P* = 0.000; *P* = 0.011) and SL-HBV (*P* = 0.000; *P* = 0.002; *P* = 0.030) groups (Fig. 1D). In addition, IL-6 expression differed between the LC and DN groups (*P* = 0.010) (Fig. 1D). IL-10 expression significantly differed between the HI group and the CHB, DN and HCC groups (*P* = 0.016; *P* = 0.021; *P* = 0.000), and the expression of IL-10 was significantly increased in the HCC group (Fig. 1E). The GRO-α concentrations in the HI and SL-HBV groups were significantly greater than those in the CHB (*P* = 0.010; *P* = 0.020), LC (*P* = 0.000; *P* = 0.000), DN (*P* = 0.001; *P* = 0.002) and HCC (*P* = 0.000; *P* = 0.001) groups (Fig. 1F). The expression of IL-8 in the CHB group was the highest, followed by that in the LC group (Fig. 1G). There were significant differences in IL-8 expression between the CHB group and the HI, SL-HBV, DN and HCC groups (*P* = 0.001; *P* = 0.011; *P* = 0.000; *P* = 0.000); moreover, the LC group and the HI, DN and HCC groups were significantly different (*P* = 0.011; *P* = 0.000; *P* = 0.000) (Fig. 1G). IP-10 was highly expressed in the CHB, LC, DN and HCC groups, and there were significant differences between these four groups and the HI (*P* = 0.000; *P* = 0.000; *P* = 0.000; *P* = 0.000) and SL-HBV (*P* = 0.004; *P* = 0.005; *P* = 0.003; *P* = 0.007) groups (Fig. 1H). However, MCP-1 expression significantly differed between the CHB and SL-HBV groups (*P* = 0.019) (Fig. 1I). These data suggest that the serum immunological markers in the SL-HBV group are similar to those in the HI group, while the CHB, LC, DN and HCC groups have different serum immunological markers due to differences in HBV activity status or disease progression. The expression of cytokines/chemokines, which are in the active phase of HBV replication, was also greater in patients with CHB or LC.

**Table 2:** Cytokine/chemokine levels of all subjects (pg/ml)

Cytokines/chemokines	HI group (*n* = 20)	HBV-infected groups				
		SL-HBV group (*n* = 11)	CHB group (*n* = 20)	LC group (*n* = 18)	DN group (*n* = 18)	HCC group (*n* = 19)
FGF-2	26.00 (24.02, 60.50)	39.58 (24.02, 118.97)	70.19 (40.58, 150.83)	34.92 (23.82, 122.25)	24.02 (8.87, 67.74)	24.02 (24.02, 44.82)
IFN-α2	0.08 (0.0125, 10.3825)	0.03 (0.01, 0.52)	21.64 (0.39, 45.46)	1.96 (0.35, 19.76)	1.38 (0.10, 36.18)	5.94 (3.42, 5.94)
IL-4	0.77 (0.38, 1.04)	1.04 (0.30, 2.47)	83.74 (18.16, 160.27)	13.52 (1.52, 134.25)	1.14 (0.40, 7.48)	1.95 (1.60, 1.95)
IL-6	2.97 (1.69, 4.39)	2.97 (1.69, 2.97)	69.78 (22.61, 261.63)	97.75 (13.58, 1852.50)	4.10 (2.97, 9.31)	55.84 (2.97, 161.98)
IL-10	2.45 (2.45, 2.45)	2.45 (2.45, 2.45)	7.27 (2.45, 25.64)	3.85 (2.45, 8.83)	6.72 (2.45, 90.46)	114.07 (2.45, 876.45)
VEGF-A	303.43 (216.07, 955.22)	547.74 (197.82, 8832.00)	530.81 (222.01, 702.97)	295.80 (78.68, 726.43)	288.83 (13.62, 5237.60)	58.12 (12.79, 273.56)
GRO-α	5031.50 (3423.25, 10607.25)	7387.00 (5929.00, 11574.00)	1459.00 (857.59, 2220.75)	571.27 (373.76, 734.56)	1112.50 (659.22, 2497.00)	1222.00 (764.34, 1462.00)
IL-8	92.23 (47.44, 201.33)	106.92 (53.18, 259.03)	2023.50 (892.65, 3674.50)	1248.00 (423.96, 3762.50)	21.46 (9.01, 55.35)	33.77 (13.04, 69.41)
IP-10	313.97 (243.00, 407.10)	349.28 (267.54, 464.90)	888.25 (461.30, 1877.00)	818.57 (594.93, 986.96)	1175.00 (550.17, 1563.25)	791.40 (676.64, 1245.00)
MCP-1	353.47 (322.68, 477.26)	322.96 (250.11, 371.92)	636.92 (437.21, 874.53)	418.52 (196.33, 848.41)	569.88 (220.36, 690.82)	600.31 (328.16, 725.10)

All data are shown as the median (Q1, Q3). FGF-2: fibroblast growth factor 2; IFN-α2: interferon-α2; IL-4: interleukin-4; IL-6: interleukin-6; IL-10: interleukin-10; VEGF-A: vascular endothelial growth factor A; GRO-α: growth-related oncogene-alpha; IL-8: interleukin-8; IP-10: interferon-γ-inducible protein 10; MCP-1: monocyte chemotactic protein 1

### Expression levels of the 10 cytokines/chemokines in HCC patients during different periods after surgical intervention

**Fig. 2:**
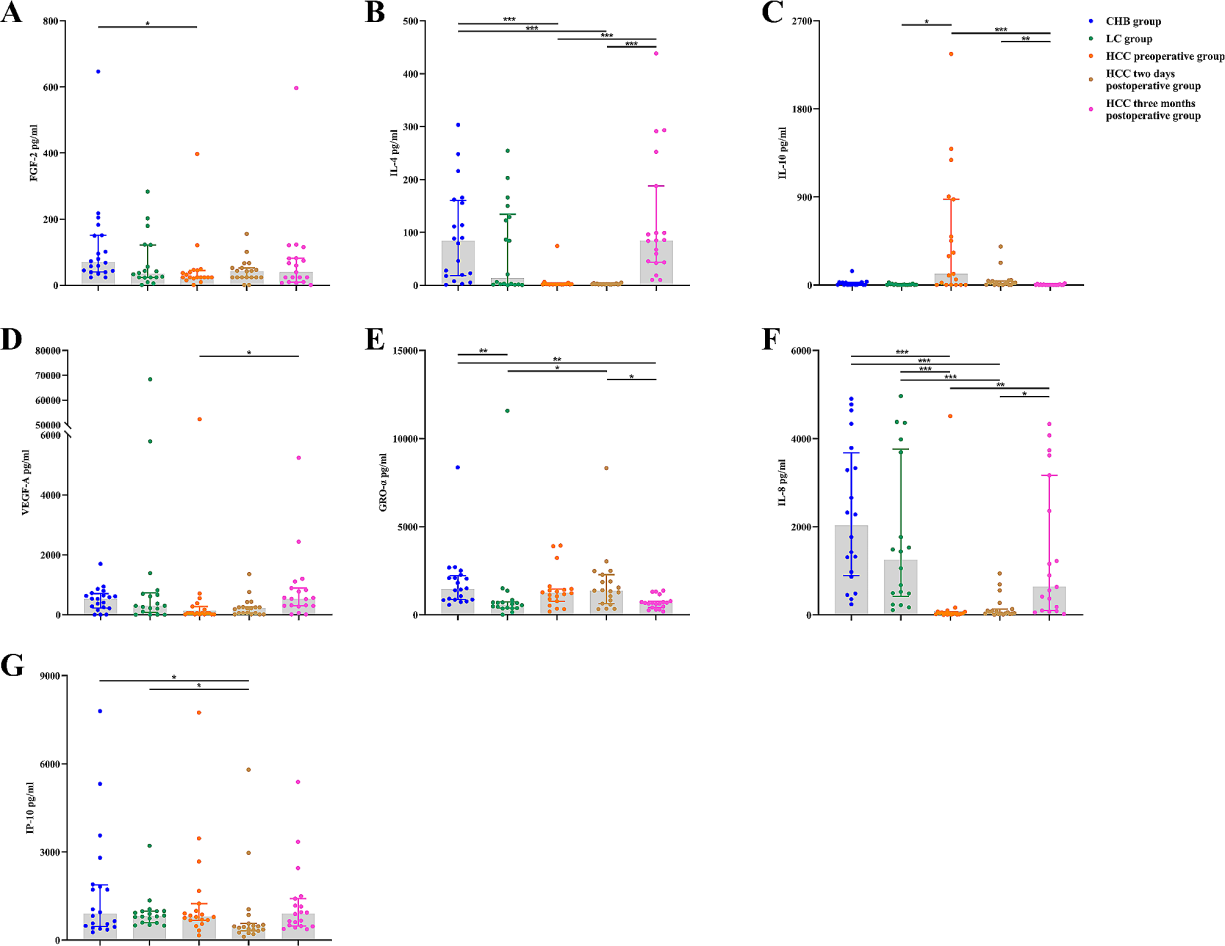
Expression of cytokines/chemokines in the CHB group, LC group, and before and after HCC surgery. * *P* < 0.05; ** *P* < 0.01; *** *P* < 0.001)

As shown in Table 3; Fig. 2, the results indicated that the serum expression levels of the 10 cytokines/chemokines were not significantly different between the preoperative group and the two-day postoperative group. However, compared with those in the other two groups, the expression levels of IL-4, VEGF-A and IL-8 tended to increase, and there was a significant difference in the 3-month postoperative group (Fig. 2B, D, F). The expression levels of IL-10 and GRO-α showed a downward trend, and there was a significant difference between the 3-month postoperative group and the other two groups (Fig. 2C, E). The expression of IP-10 decreased in the 2-day postoperative group but increased in the preoperative and 3-month postoperative groups (Fig. 2G).

**Table 3:** Cytokine/chemokine levels in preoperative and postoperative HCC periods (pg/ml)

Cytokines	HCC periods		
	HCC preoperative group	HCC two days postoperative group	HCC three months postoperative group
FGF-2	24.02 (24.02, 44.82)	42.23 (24.02, 52.39)	39.60 (9.50, 81.83)
IFN-α2	5.94 (3.42, 5.94)	5.94 (3.42, 5.94)	5.94 (3.42, 10.52)
IL-4	1.95 (1.60, 1.95)	1.95 (1.60, 1.95)	84.47 (43.26, 187.56)
IL-6	55.84 (2.97, 161.98)	72.27 (15.44, 136.27)	56.54 (20.78, 347.25)
IL-10	114.07 (2.45, 876.45)	24.40 (2.93, 42.85)	2.45 (2.45, 5.97)
VEGF-A	58.12 (12.79, 273.56)	214.05 (58.12, 254.07)	527.52 (295.85, 885.90)
GRO-α	1222.00 (764.34, 1462.00)	1360.00 (631.64, 2269.00)	664.84 (412.62, 769.93)
IL-8	33.77 (13.04, 69.41)	58.60 (26.00, 132.98)	634.32 (100.71, 3169.00)
IP-10	791.40 (676.64, 1245.00)	449.34 (315.43, 565.65)	888.55 (477.99, 1410.00)
MCP-1	600.31 (328.16, 725.10)	432.06 (275.12, 735.51)	489.68 (405.08, 682.12)

All data are shown as the median (Q1, Q3)

Almost all HBV-related HCCs in China evolve from LC, and the patients with HCC in this study also had a background of LC. Therefore, the differences among the preoperative HCC group, 2-day postoperative group, 3-month postoperative CHB group and LC group were further analyzed. In the preoperative HCC group, the FGF-2, IL-4, and IL-8 levels were significantly lower than those in the CHB group (Fig. 2A, B, F); the IL-8 expression was also significantly lower than that in the LC group (Fig. 2F); and the IL-10 expression was significantly greater than that in the LC group (Fig. 2C). In the 2-day postoperative group, the IL-4, IL-8, and IP-10 levels were significantly lower than those in the CHB group (Fig. 2B, F, G); the IL-8 and IP-10 levels were also significantly lower than those in the LC group (Fig. 2F, G); and the GRO-α expression was significantly greater than that in the LC group (Fig. 2E). For the 3-month postoperative group, only the GRO-α level was lower than that in the CHB group (Fig. 2E). The above data indicate that the specific cytokine changes observed in the preoperative (tumor-bearing) group might be closely related to HCC, and the markedly elevated IL-10 and markedly decreased IL-8 levels in at least some HCC patients might be related to HCC progression. The immunological status of the 3-month postoperative group who had not relapsed after resection was similar to that of the CHB and LC patients, mainly in terms of the inflammatory phenotype, as they no longer exhibited a phenotype caused by cancer cells.

### Correlation analysis of the 10 cytokines/chemokines in HCC patients during different periods after surgical intervention

**Fig. 3:**
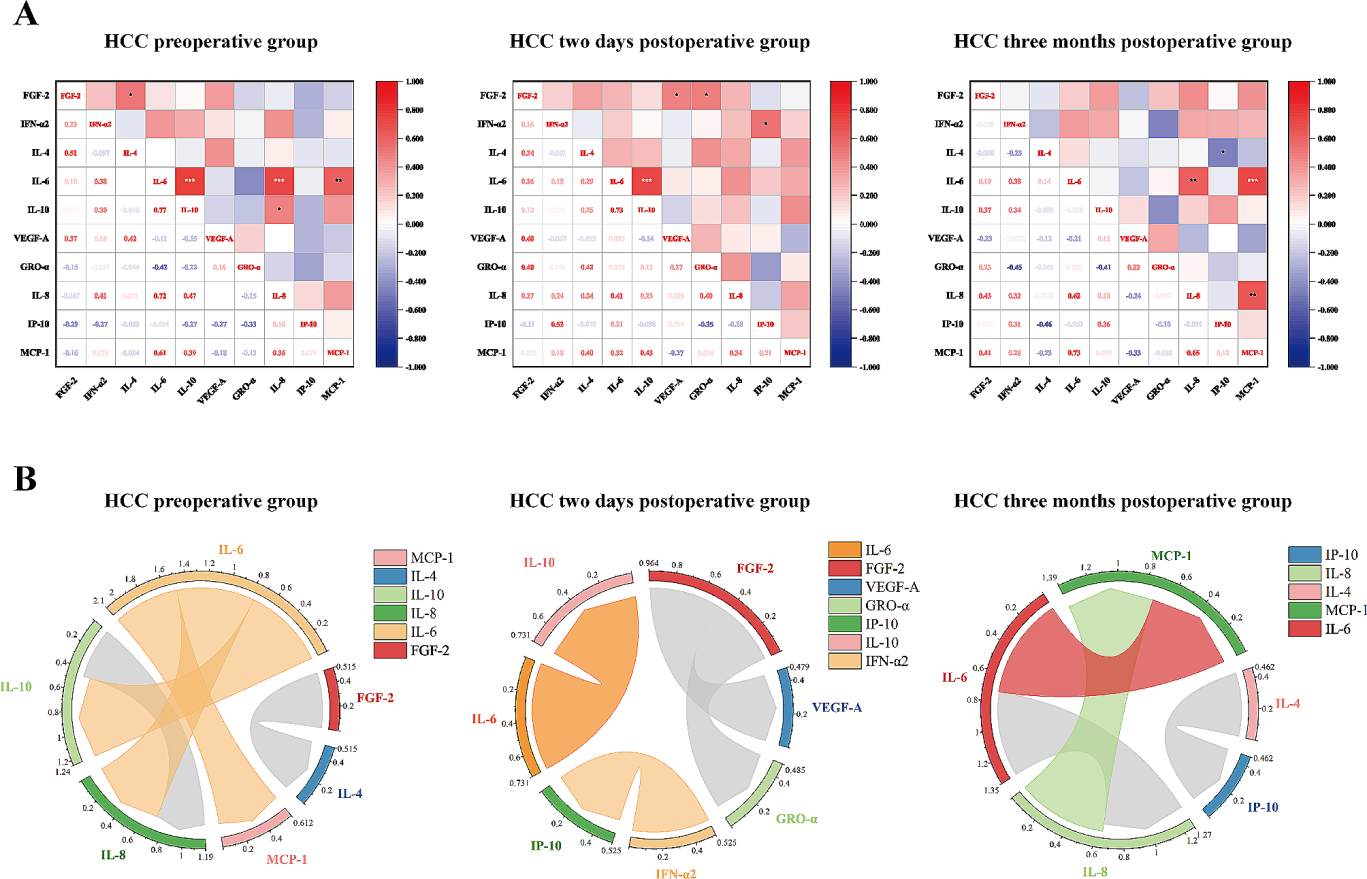
Correlation analysis of cytokines/chemokines in the preoperative and postoperative HCC periods. Correlation analysis of FGF-2, IFN-α2, IL-4, IL-10, VEGF-A, GRO-α, IL-8, IP-10, and MCP-1 in the HCC preoperative, HCC two-day postoperative and HCC three-month postoperative groups (**A**). Circos plots were used to illustrate the correlation networks of the 10 cytokines/chemokines in the preoperative and postoperative HCC periods, and only significant correlations (*P* < 0.05) are shown (**B**). (* *P* < 0.05; ** *P* < 0.01; *** *P* < 0.001)

Studies have shown complex interactions and significant differences in cytokine/chemokine levels at different periods of HCC treatment relative to the time of surgery. Most of the cytokines/chemokines with significant correlation differences were positively correlated both before HCC and after HCC (Fig. 3), implying that the increases in these cytokines/chemokines were mainly accompanied by increases in the levels of other inflammatory molecules. Notably, IL-6 was the marker most closely linked to the preoperative HCC group, and IL-10, IL-8 and MCP-1 contributed the most to this increase (*r* = 0.767, *P* = 0.000; *r* = 0.720, *P* = 0.001; *r* = 0.612, *P* = 0.005; respectively); there was also a positive correlation between IL-10 and IL-8 (*r* = 0.473, *P* = 0.041). In addition, FGF-2 was positively correlated with IL-4 (*r* = 0.515, *P* = 0.024). At 2 days after HCC surgery, IL-10 expression decreased sharply, and IL-6 and IL-10 were strongly positively correlated (*r* = 0.731, *P* = 0.000). FGF-2 was positively correlated with VEGF-A and GRO-α (*r* = 0.479, *P* = 0.038; *r* = 0.485, *P* = 0.035; respectively); IFN-α2 was positively correlated with IP-10 (*r* = 0.525, *P* = 0.021). However, the increase in IL-8 expression was closely related to the increase in IL-6 and MCP-1 at 3 months after HCC surgery (*r* = 0.617, *P* = 0.005; *r* = 0.653, *P* = 0.002; respectively), and the increase in IL-6 and MCP-1 was strongly correlated (*r* = 0.733, *P* = 0.000). However, IP-10 was negatively correlated with IL-4 (*r* = -0.462, *P* = 0. 047).

### Correlations between the 10 cytokines/chemokines and clinical data

**Fig. 4:**
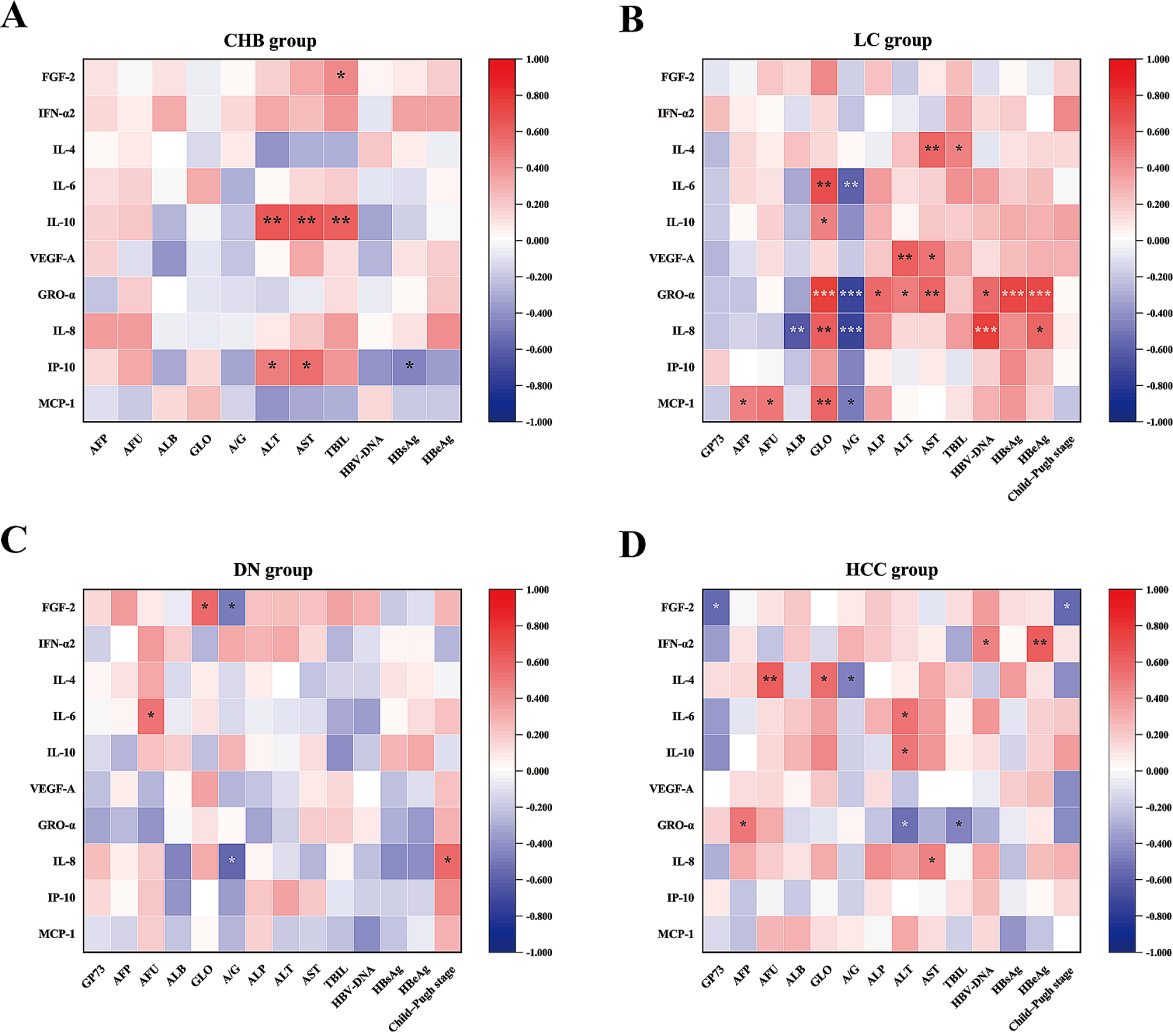
Correlations between the 10 cytokines/chemokines and clinical data. (* *P* < 0.05; ** *P* < 0.01; *** *P* < 0.001)

In the CHB group, FGF-2 was positively correlated with TBIL (*r* = 0.451, *P* = 0.046); IL-10 was positively correlated with ALT, AST and TBIL (*r* = 0.640, *P* = 0.002; *r* = 0.647, *P* = 0.002; *r* = 0.612, *P* = 0.004; respectively) (Fig. 4A); and IP-10 was positively correlated with ALT and AST (*r* = 0.494, *P* = 0.027; *r* = 0.547, *P* = 0.013; respectively) but negatively correlated with HBsAg (*r* = -0.460, *P* = 0.041) (Fig. 4A).

In the LC group, the chemokine GRO-α had the closest correlation with the clinical data. GRO-α was positively correlated with GLO, ALP, ALT, AST, HBV-DNA, HBsAg and HBeAg (*r* = 0.775, *P* = 0.000; *r* = 0.577, *P* = 0.012; *r* = 0.488, *P* = 0.040; *r* = 0.593, *P* = 0.009; *r* = 0.564, *P* = 0.015; *r* = 0.730, *P* = 0.001; and *r* = 0.719, *P* = 0.001, respectively) but negatively correlated with A/G (*r* = -0.722, *P* = 0.001) (Fig. 4B). In addition, IL-8 was positively correlated with GLO, HBV-DNA and HBeAg (*r* = 0.624, *P* = 0.006; *r* = 0.779, *P* = 0.000; and *r* = 0.581, *P* = 0.011, respectively) but negatively correlated with ALB and A/G (*r* = -0.631, *P* = 0.005; and *r* = -0.740, *P* = 0.000, respectively) (Fig. 4B). MCP-1 was positively correlated with AFP, AFU and GLO (*r* = 0.474, *P* = 0.047; *r* = 0.517, *P* = 0.028; and *r* = 0.595, *P* = 0.009, respectively) but negatively correlated with A/G (*r* = -0.489, *P* = 0.040) (Fig. 4B). IL-4 was positively correlated with AST and TBIL (*r* = 0.539, *P* = 0.009; *r* = 0.480, *P* = 0.044; respectively), while IL-6 was positively correlated with GLO (*r* = 0.688, *P* = 0.002) but negatively correlated with A/G (*r* = -0.591, *P* = 0.010) (Fig. 4B). VEGF-A was positively correlated with ALT and AST (*r* = 0.603, *P* = 0.008; *r* = 0.530, *P* = 0.024, respectively) (Fig. 4B).

In the DN group, FGF-2 was positively correlated with GLO (*r* = 0.575, *P* = 0.012) but negatively correlated with A/G (*r* = -0.481, *P* = 0.043) (Fig. 4C). IL-6 was positively correlated with AFU (*r* = 0.521, *P* = 0.027) (Fig. 4C). IL-8 levels were positively correlated with Child‒Pugh stage (*r* = 0.565, *P* = 0.015) but negatively correlated with A/G (*r* = -0.569, *P* = 0.014) (Fig. 4C).

In the HCC group, FGF-2 was negatively correlated with GP73 and Child‒Pugh stage (*r* = -0.542, *P* = 0.017; *r* = -0.546, *P* = 0.016, respectively) (Fig. 4D). IFN-α2 was positively correlated with HBV-DNA and HBeAg (*r* = 0.470, *P* = 0.042; and *r* = 0.634, *P* = 0.004, respectively) (Fig. 4D). IL-4 was positively correlated with AFU and GLO (*r* = 0.627, *P* = 0.004; *r* = 0.555, *P* = 0.014, respectively) but negatively correlated with A/G (*r* = -0.479, *P* = 0.038) (Fig. 4D). IL-6 was positively correlated with ALT (*r* = 0.523, *P* = 0.022). IL-10 was positively correlated with ALT (*r* = 0.502, *P* = 0.029) (Fig. 4D). GRO-α was positively correlated with AFP (*r* = 0.511, *P* = 0.026) but negatively correlated with ALT and TBIL (*r* = -0.538, *P* = 0.018; and *r* = -0.458, *P* = 0.049, respectively) (Fig. 4D). IL-8 was positively correlated with AST (*r* = 0.474, *P* = 0.040) (Fig. 4D).

## Discussion

HBV poses a severe threat to public health and is a high risk factor for chronic hepatitis, occult hepatitis, cirrhosis, and HCC [1, 14]. As HBV is a noncytopathic virus, the associated inflammation and direct liver damage caused by this virus are mediated by the immune response [9]. Understanding the expression levels of serum cytokines is crucial for understanding the development and metastasis of tumors and is helpful for determining the prognosis and treatment of tumors [7, 15, 16]. Traditional enzyme-linked immunosorbent assays (ELISAs) usually detect only one cytokine at a time [16]. Therefore, Luminex liquid chip analysis was used in this study because this technology has high sensitivity and can simultaneously detect ten serum cytokines associated with HBV infection [11]. In addition, compared with solid-phase chip technology, the Luminex liquid chip has the advantages of a simple procedure, good repeatability, and high throughput [9, 17].

Host immune factors play important roles in the outcome of HBV infection. A natural complete response to HBV leads to self-healing, while an inadequate or deficient immune response may lead to persistent viral replication and necrotic liver inflammation, resulting in chronic HBV infection, cirrhosis, and even HCC [9]. Cytokine/chemokine homeostasis plays a key role in the occurrence and development of hepatitis B. Abundant cytokines/chemokines not only participate in the initiation and regulation of the immune response but also directly or indirectly participate in the inhibition of viral replication and even malignant liver cell transformation [9, 16]. Multiple cytokines are differentially expressed in patients with CHB, LC or HCC, suggesting that the cytokine profile may have certain diagnostic and prognostic value [2, 6, 7, 16, 18, 19]. However, there are great differences among different researchers and among different detection methods. Moreover, there is a lack of dynamic analyses of liver disease progression at different stages and of HCC before and after surgery. Therefore, serum samples were collected from patients with CHB or HBV-related LC, DNs or HCC before and after HCC surgery for analysis; healthy controls and patients with SL-HBV infection were used as controls. The results showed that CHB patients with persistent HBV infection had a clear inflammatory biomarker profile and had the highest level of immune disorders, followed by patients with cirrhosis. Among the studied biomarkers, 4 cytokines (FGF-2, IFN-α2, IL-4 and IL-6) and 3 chemokines (IL-8, IP-10 and MCP-1) were highly expressed in the CHB group, 2 cytokines (IL-4 and IL-6) and 2 chemokines (IL-8 and IP-10) were highly expressed in the LC group, and only IP-10 and IL-10 were highly expressed in the DN group and the HCC group, respectively.

According to their receptors and roles in tumorigenesis and development, cytokines/chemokines can be divided into three categories. The cytokines/chemokines associated with the inflammatory response include IFN-α2 [20], IL-4 [21], IL-6 [22], IL-10 [23], IL-8 [24] and IP-10 [25], and IL-6 is an important proinflammatory cytokine. IFN-α2, IL-4 and IL-10 are important anti-inflammatory cytokines. IL-8 and IP-10 are chemokines that induce the migration of immune cells to inflamed tissues. Angiogenesis-related cytokines/chemokines include FGF-2 [26], VEGF-A [27] and GRO-α [28]. MCP-1 is a chemokine associated with tumor growth, invasion, and metastasis [29]. The functions of cytokines are multifaceted, and they can participate in the regulation of different signaling pathways in different immune microenvironments and even have bidirectional regulatory functions, such as proinflammatory/anti-inflammatory and procancer/anticancer functions [3, 9, 10]. 

During HBV infection, especially in CHB patients, IFN-α2 is a vital cytokine for immune stimulation and viral clearance, and an increase in IFN-α2 is related to activation of the anti-HBV immune response [18]. IFN-α expression is significantly increased in patients with CHB and might inhibit HBV replication by inhibiting the transcriptional activity of covalently closed circular DNA (cccDNA) [30]. The results of this study confirmed that IFN-α2 was highly expressed in the CHB and HCC groups, with both of these groups exhibiting significantly greater IFN-α2 levels than the HI and SL-HBV groups.

Previous studies have shown that IL-4 plays dual roles in HBV infection. An increase in the IL-4 level in the peripheral blood can inhibit the production of IFN-γ and induce a Th1-type immune response, leading to continuous HBV replication and promoting the establishment of immune tolerance [31]. Paradoxically, IL-4 also inhibits HBsAg production and HBV replication, thereby mediating the host antiviral response [10]. In this study, IL-4 was highly expressed in the CHB and LC cohorts, which indicated that the CHB and LC cohorts were in a period of active viral replication and a strong antiviral response.

IL-6 is an inflammatory cytokine produced by macrophages that promotes tumor growth and progression by promoting chronic inflammation [22]. In HBV infection, on the one hand, IL-6 can eliminate the virus by stimulating an immune response via infected hepatocytes; on the other hand, it can induce the occurrence of hepatitis, LC and HCC [14]. In this study, the IL-6 levels in the CHB, LC and HCC groups were significantly greater than those in the HI and SL-HBV groups, which also confirmed the findings of the above study.

IL-10 is a multifunctional immunosuppressive cytokine secreted by Th2 cells, cancer cells and other cells and is an important cytokine that downregulates the activity of other immune cells and leads to persistent infection [3, 31]. Chau GY et al. reported that tumor cells secreted IL-10 and that the level of IL-10 decreased significantly after HCC resection [15]. Further research showed that the serum IL-10 concentration was a negative prognostic factor for a significantly shortened median survival time [32, 33]. Therefore, in the present study, the high expression of IL-10 in the HCC group and its rapid decrease after surgery suggested that IL-10 can be secreted by cancer cells, and its dynamic changes before and after surgery can be used for prognostic evaluation. In contrast to the above results, the IL-10 level measured by Su Z et al. was significantly lower in the hepatoma group than in the CHB group [34].

IL-8, a proinflammatory CXC chemokine, is significantly elevated in the serum of CHB and HBV-associated HCC patients [35, 36]. However, the results of this study showed that IL-8 was highly expressed in the CHB and LC groups, with significantly greater levels in those groups than in the HCC group. However, the IL-8 concentration significantly increased 3 months after surgery, and these changes need to be further confirmed in HCC patients.

According to the analysis of the three groups before and after HCC surgery, the expression levels of IL-4, VEGF-A and IL-8 tended to increase, and the expression levels of IL-10 and GRO-α tended to decrease, suggesting that these 5 cytokines/chemokines may be closely related to HCC progression or prognosis. It was speculated that these factors may be useful in evaluating patient prognosis after HCC surgery. Similarly, Chen et al. used preoperative serum to select 6 out of 39 cytokines/chemokines to construct a novel cytokine-based prognostic classifier (CBPC) to predict HCC recurrence [16]. The levels of 4 cytokines/chemokines (GRO, IL-8, IP-10 and VEGF) were consistent with the findings of this paper. Jekarl et al. analyzed the changes in 13 cytokine profiles in HCC patients treated with transarterial chemoembolization (TACE). This study indicated that TACE increased or decreased the levels of most cytokines, including the three cytokines also studied in this paper, within 60 days after treatment. The change in IL-10 was consistent with that reported in this paper, in which a decreasing trend was shown, while the IL-6 level increased and the IL-4 level remained stable [6]. Therefore, it is speculated that the differential expression of cytokines/chemokines may reflect the therapeutic differences between radical resection and TACE.

Analysis of the correlations of the 10 cytokines/chemokines in the three periods before and after HCC surgery revealed that most of the cytokines/chemokines with significant correlation differences were positively correlated before and after surgery, suggesting that increases in the levels of these cytokines/chemokines might be accompanied by increases in other inflammatory molecules. Vinhaes et al. used molecular degree of perturbation (MDP) scores to assess global inflammatory imbalances associated with unitary HBV infection and found that the significant associations of most factors were positive despite the use of different clinical groups [14]. That is, increased expression of a specific marker is usually accompanied by increased levels of perturbations in other inflammatory molecules, especially in chronic HBV carriers. Ribeiro et al. also reported positive correlations between IFN-γ, TNF-α, IL-10, IL-6, IL-4, and IL-2 in HBV-positive individuals, with the levels of these cytokines tending to increase together [37].

Correlation analysis between cytokines/chemokines and clinical indicators revealed that in the CHB, LC, DN and HCC groups, there were 6 pairs of positive correlations and 1 pair of negative correlations; 18 pairs of positive correlations and 5 pairs of negative correlations; 3 pairs of positive correlations and 2 pairs of negative correlations; and 8 pairs of positive correlations and 5 pairs of negative correlations, respectively. Although no consistency or regular change in any index was found among the groups due to the large number of indicators, small sample size, or complexity of the factor network, similar research results were obtained in similar studies [13, 19, 25, 34]. Lian et al. reported that, compared with those in the HI and SL-HBV groups, the serum IL-10 and IP-10 concentrations in the CHB patient group were increased and positively correlated with ALT levels [13]. Wiegand et al. reported the highest concentration of CXCL10 (IP-10) in hepatitis patients (the HBeAg-positive hepatitis phase [EPH]), and this concentration significantly differed from that in HBV carriers (inactive carriers [ENIs]). Furthermore, IP-10 concentrations showed the strongest correlation with ALT titers in EPH (*r* = 0.53, *P* < 0.001) and ENH patients [19]. Su Z et al. showed significantly elevated levels of IP-10 in CHB patients with abnormal liver function, and IP-10 was positively correlated with ALT and AST levels [34]. These results suggest that CXCL10 (IP-10) may be a useful marker for further characterizing the stage of chronic HBV infection.

This study has several potential limitations. First, the sample size was small, and the number of time points for dynamic monitoring was also small. In addition, cytokine levels might be affected by other diseases, anti-inflammatory drugs, or individual differences. Moreover, this study focused on only 10 circulating cytokines and chemokines. Finally, enzyme-linked immunospot (ELISPOT) assays were not used to detect HBV-specific T-cell responses in the peripheral circulation.

## Conclusion

In summary, in this study, 10 cytokines/chemokines were selected for further exploration of their serum expression in patients with HBV infection-related diseases, and dynamic fluctuations in their levels were observed during the preoperative and postoperative periods in HCC patients. Moreover, there were no significant differences in cytokine/chemokine levels between patients with SL-HBV infection and healthy people. Increases in the levels of IL-4, IL-6, IL-8 and IP-10 mediated inflammatory immunity in patients with CHB or LC. A high expression level of IL-10 in the tumor microenvironment might be involved in the progression of HCC. The significant increases in IL-4, VEGF-A and IL-8 expression and significant decreases in IL-10 and GRO-α expression at 3 months after surgery might be useful for evaluating the prognosis of HCC patients. Therefore, this study confirmed that specific cytokine phenotypes are closely related to the occurrence of liver disease and the progression of patients with liver disease and that screening multiple combinations of circulating cytokines can be used as a new screening method for the assessment of liver disease progression.

## Data Availability

The datasets used and analyzed during the current study are available from the corresponding author upon reasonable request.
